# The clinical significance of CTC enrichment by GPC3-IML and its genetic analysis in hepatocellular carcinoma

**DOI:** 10.1186/s12951-021-00818-3

**Published:** 2021-03-16

**Authors:** Bin Yi, Tian Wu, Nan Zhu, Yao Huang, Xiaoyu Yang, Lei Yuan, Yingjun Wu, Xiaofei Liang, Xiaoqing Jiang

**Affiliations:** 1 Department of Organ Transplantation, Eastern Hepatobiliary Surgery Hospital, Second Military Medical University, Shanghai, China; 2 Jukang (Shanghai) Biotechnology Co. Ltd., 28, Xiangle Rd., Shanghai, 201800 China; 3 Department I of Biliary Tract, Eastern Hepatobiliary Surgery Hospital, Second Military Medical University, No. 225, Changhai Rd., Shanghai, 200438 China

**Keywords:** Hepatocellular carcinoma (HCC), Circulating tumor cells (CTCs), Glypican-3 (GPC-3), GPC3 immunoliposomes (GPC3-IML), Next generation sequencing (NGS)

## Abstract

**Background:**

This research was to develop a special method for enriching Circulating tumor cells (CTCs) of Hepatocellular carcinoma (HCC) by Glypican-3 immunoliposomes (GPC3-IML), and to analyze the correlation between the CTCs count and tumor malignancy, as well as to investigate the mutation characteristics of CTC-derived NGS.

**Results:**

In this study characterization of physical parameters was performed with the preparation of GPC3-IML. CTCs in peripheral blood of HCC patients were further separated and identified. Immunofluorescence was used to identify CTCs for further counting. By this means, the correlation between CTCs count and clinicopathological features was analyzed, and the genetic mutation characteristics of NGS derived from CTCs were investigated and compared with that of tissue NGS. Results showed that compared with EpCAM and vimentin, GPC-3 had a stronger CTCs separation ability. There was a correlation between "positive" count of CTCs (≥ 5 PV-CTC per 7.5 ml blood) and BCLC stage (*P* = 0.055). The result of CTC-NGS was consistent with that of tissue-NGS in 60% cases, revealing that *KMT2C* was a common highly-frequent mutated gene.

**Conclusion:**

The combination of immunomagnetic separation of CTCs and anti-tumor marker identification technology can be regarded as a new technology of CTCs detection in peripheral blood of patients with HCC.

*Trial registration* EHBHKY2020-k-024. Registered 17 August 2020—Retrospectively registered
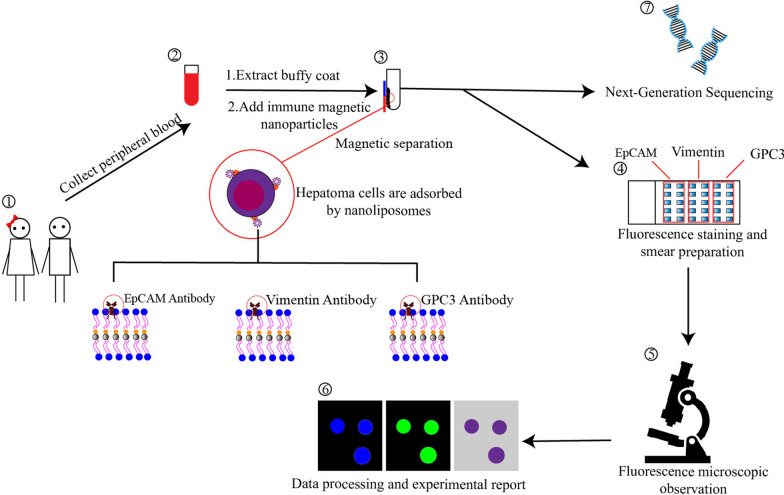

## Background

Metastasis of hepatocellular carcinoma (HCC) is the consequence of tumor cell that disperse from the primary focus, travel into the blood, and then invade and colonize new sites [1]. Nowadays, technologies can effectively detect the circulating tumor cells (CTCs) that released into the blood, and the standard method used in clinical is CellSearch system based on EpCAM antibody coated magnetic particles, which is used to detect the content of CTCs in colon cancer and prostate cancer [2, 3]. Fang et al. [4] enumerate CTCs in the right atrium and peripheral blood of patients in the perioperative period of TACE using immunomagnetic EpCAM method. The results showed that CTC count in the right atrium was higher than that in the peripheral blood. In another study carried out by Sun et al. [5], they counted the CTCs of 123 HCC patients treated with resection both preoperatively and postoperatively, and found that patients with preoperative CTCs ≥ 2 in 7.5 ml blood showed an earlier recurrence risk than those with CTCs < 2. CTCs ≥ 2 was revealed to be an independent predictor of tumor recurrence. Furthermore, it has been reported that CTCs in the blood of patients with HCC correlate directly with prognostic factors, such as overall survival (OS), progression free survival, and tumor stage. For example, Schulze et al. [6] found that the OS of patients with EpCAM^+^ CTCs was shorter than that of patients without. CTC showed a positive correlation with Barcelona-Clinic Liver Cancer (BCLC) stage, suggesting that positive CTCs may predict the prognosis of patients with HCC. Meanwhile, Nel et al. [7] revealed that insulin-like growth factor binding protein (IGFBP)-1 mRNA level was correlated with the time to progression (TTP) by detecting CTCs in HCC patients undergone selective internal radiotherapy (SIRT), supporting that CTCs can be a new molecular marker for individualized treatment of HCC. In addition, Chen et al. [8] carried out a retrospective analysis in terms of the level and classification of CTCs in 195 HCC patients. It was concluded that the total CTC count was related to BCLC stage, tumor metastasis and serum AFP level. Besides, patients with recurrence had higher mixed-and interstitial type of CTCs [8]. It implied that CTC count and epithelial-mesenchymal transition (EMT) classification of CTC were potentially associated with the prognosis of HCC patients. [8]

Considering the heterogeneity of HCC and the occurrence of EMT in the process of metastasis, there may be limitation in the use of epithelial cell marker EpCAM to detect CTCs. In the study performed by Xu et al. [9], 69 patients with positive CTCs were successfully screened from 85 patients with HCC by using specific ligand of asialoglycoprotein receptor to wrap magnetic particles. Meanwhile, MXR7 (GPC3) cDNA has a high positive rate in tumor tissues of patients with HCC [10]. Abundant tissue microarray and immunohistochemistry data suggested that GPC3 (a subtype of HSPG) protein expressed highly in over 70% of HCC tumor samples, but none in normal liver tissue, benign liver disease, liver cirrhosis and hepatitis tissues [11–13]. Besides, the expression of GPC3 exhibited a certain correlation with the prognosis of patients [14, 15]. So far, MRI, PET and NIR images targeting GPC3 have been applied in clinical practice for early detection of HCC [16–19]. A variety of immunotherapy programs have been developed for GPC3 + solid tumors. Among them, some programs are based on anti-GPC3 antibodies (monoclonal antibodies, peptide vaccines, immunotoxins, bispecific antibodies, etc.), some ones are GPC3-targeted CAR-T/NK therapies, and some have entered phase I/II clinical trials [20–23].

In this study, CTCs in peripheral blood of HCC patients were enriched by innovative multi-site immunoliposomes (IML) for immunofluorescence identification, were labeled by GPC3 (a subtype of HSPG) antibody-coated magnetic particles, and then were separated immunomagnegically. Through this system, separated CTCs were applied for next generation sequencing to explore the genomic alterations. The relations between CTCs features and clinicopathological factors were analyzed.

## Results

### GPC3 CTC single-cell separation and gene analysis method

**Fig. 1:**
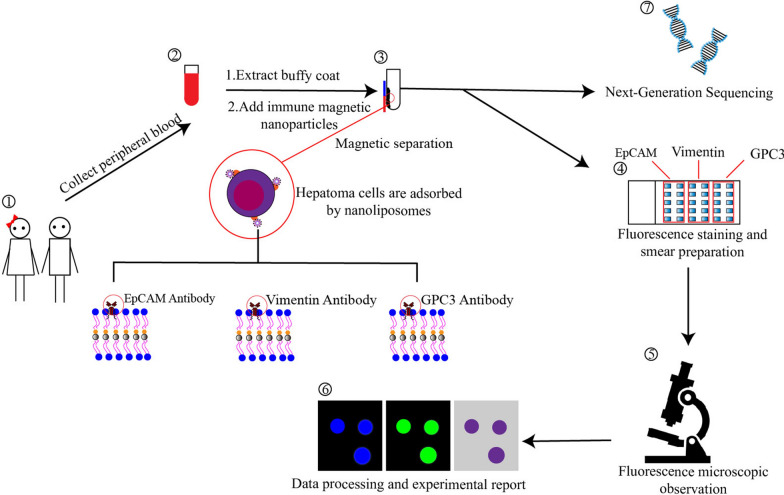
Preparation process of IMLs and flow chart of CTC separation and identification

GPC3-CTC single-cell separation and gene analysis process mainly included four processes as follows. Firstly, the supernatant of peripheral blood samples were collected and centrifuged at 1000*g* for 10 min. Secondly, the buffy coat was taken out, and magnetic separation was performed after the addition of magnetosphere loaded with different antibodies. Thirdly, immunofluorescence staining was carried out in one part of the separated CTCs, and the results were observed under fluorescence microscope for further statistical analysis. Finally, the rest of the separated CTCs were used for next generation sequencing (CTC-NGS) which results were compared with the sequencing results in tumor tissue (Fig. 1).

The established CTC capture platform has two important functional features. Specifically, the separated tumor cells with different phenotypes can reach the single-cell level on a special slide, which would facilitate subsequent study of heterogeneity. Besides, a comparison of CTC blood sample with HCC tissue can highlight more significant clinical value of the CTC study.

### Performance evaluation of GPC3-CTC separation and detection system

**Fig. 2:**
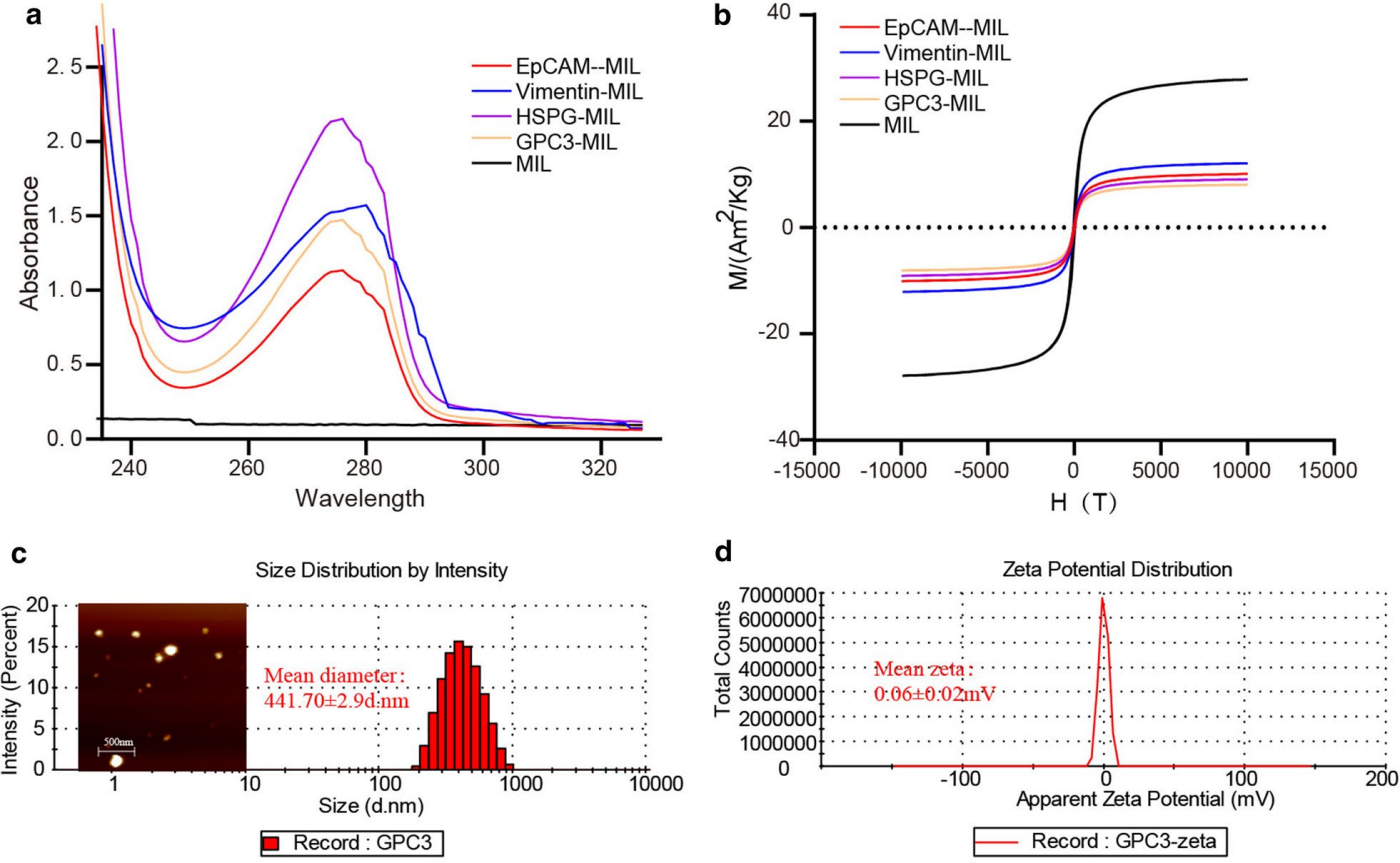
Characterization and particle size distribution of IML. **a** Ultraviolet absorption spectrum of nano-immunoliposomes coated with EpCAM (Red), vimentin (Blue), GPC3 (Orange), or HSPG (Purple). **b** Magnetic saturation curve of nano-immunoliposomes coated with EpCAM (Red), vimentin (Blue), GPC3 (Orange), or HSPG (Purple). **c** Particle-size-distribution based intensity of GPC3-IML. **d** Zeta potential of GPC3-IML

In GPC3-CTC separation and detection system, the GPC3-positive magnetic nanoparticles would exert great impact on the separation efficiency of GPC3-positive cells in blood. As shown in Fig. 2a, the obvious UV absorption peak around 280 nm was observed in nano-immunoliposomes coated with EpCAM, vimentin, or GPC3 antibodies respectively. It suggested that modified antibodies appeared on the surface of nano-immunoliposomes, and its content was controllable. Figure 2b shows the magnetic saturation curve of GPC3-IML and other immunoliposomes. The results revealed that the prepared nano-immunoliposomes (EpCAM, Vimentin and GPC3) had high saturation magnetizationas well as superparamagnetism. At 300 k, the saturation magnetization of GPC3-IML was 30.97 emu/g, about 68.4% of that of Fe_3_O_4_ naked immunoliposomes. Furthermore, the particle size distribution of GPC3-IM was shown in Fig. 2c, with an average particle size of 441.70 ± 2.9 nm, which was similar to that shown in the image photographed by AFM. In addition, GPC3-IML had a surface potential of 0.06 ± 0.02 mV and was neutral, which can reduce the interference from the non-specific adsorption caused by positive current reported in previous studies (Fig. 2d). The TEM images of GPC3-IML, EpCAM-IML and Vimentin-IML were displayed in Additional file 1: Figure S1.

### The capture efficiency of GPC3-IML

**Fig. 3:**
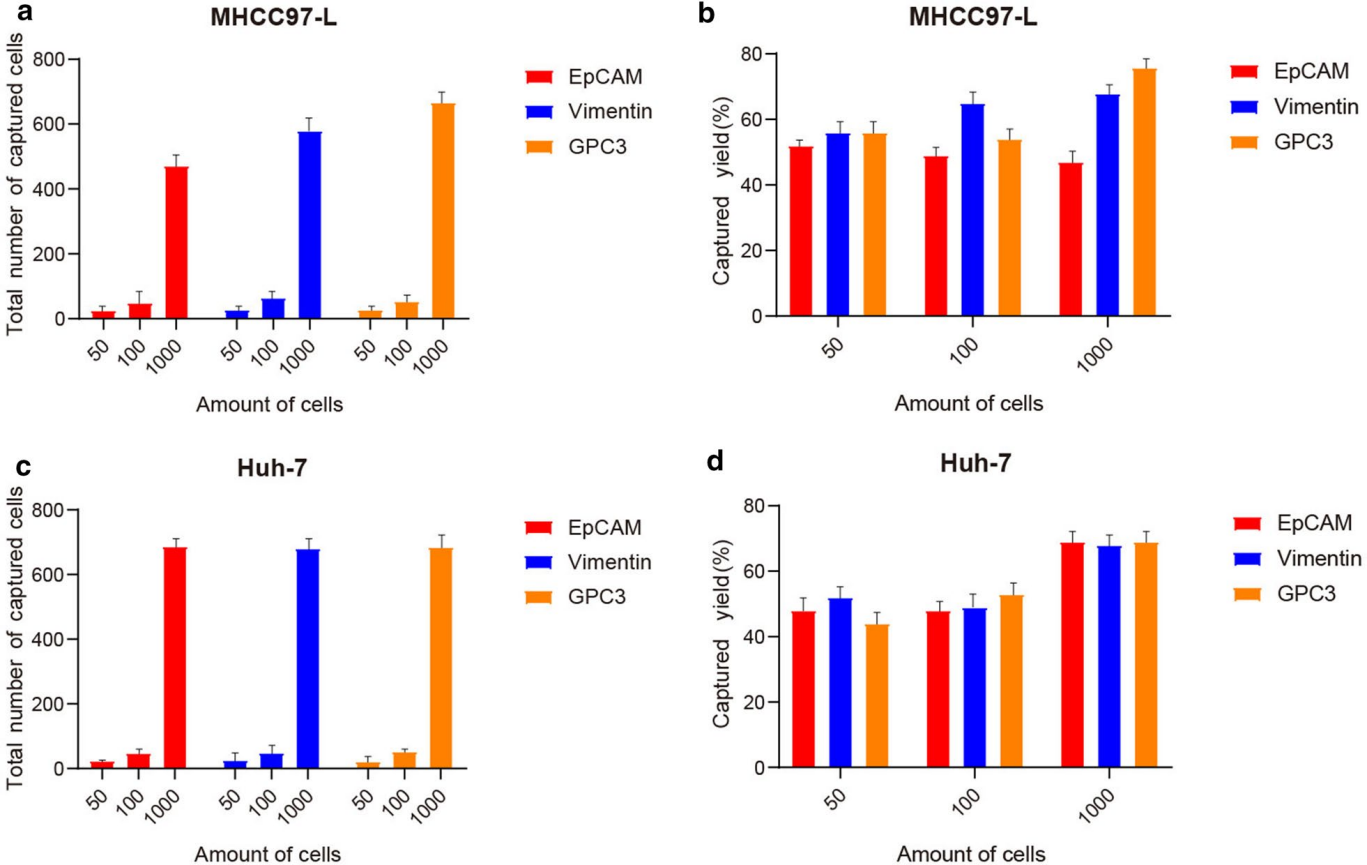
Capture efficiency of IMLs. The total captured cell numbers of EpCAM-IML (Red), Vimentin-IML (Blue) and GPC3-IML (Orange) under the cell concentration 50 cells/mL, 100 cells/mL and 1000 cells/mL in MHCC97-L cell line (**a**) and Huh-7 cell line (**c**). The capture yield of EpCAM-IML (Red), Vimentin-IML (Blue) and GPC3-IML (Orange) under the cell concentration 50 cells/mL, 100 cells/mL and 1000 cells/mL in MHCC97-L cell line (**b**) and Huh-7 cell line (**d**)

In order to verify the capture efficiency of GPC3-IML, the nano-immunoliposomes coated with EpCAM, Vimentin and GPC3 were tested on two GPC3-positive HCC cell lines (MHCC97-L cell line and Huh-7 cell line). As shown in Fig. 3a and c, three concentrations of 50 cells/mL, 100 cells/mL and 1000 cells/mL were selected, and the total number of captured cells also showed an increased trend along with the increase of concentration in the tests for three types of nano-immunoliposomes. Superb cell capture efficiency (> 70%) was accomplished using the GPC3-IML. At the cell concentration of 1000 cells/mL, the capture efficiency of GPC3-IML in two GPC3-positive HCC cell lines was slightly different, for MHCC97-L cell line presented a higher capture efficiency (Fig. 3b and d). In addition, compared with the conventional EpCAM-IML and Vimentin-IML, GPC3-IML showed an excellent capture efficiency.

### Targeted recognition ability of GPC3-IML to hepatoma cells

High biocompatibility and low cytotoxicity are accepted as the preconditions for the wide application of biomaterials. As shown in Additional file 2: Figure S2A-2C, the inhibition rate of GPC3-IML constructed in this experiment was low in HCC cells at the concentration below 100 μg/mL. The cytotoxicity of GPC3-IML to HCC cells gradually increased when the concentration was higher than 200 μg/mL. The results of Additional file 2: Figure S2D revealed that there was no significant difference among cell lines about the inhibition ability of the constructed IML, and it was only associated with the concentration of immunoliposomes. Therefore, the low toxicity of the constructed IML to the captured cells in normal use laid the foundation for CTC culture, surface marker analysis, cell behavior analysis and gene analysis.

### The interaction between GPC3-IML and HCC CTCs

**Fig. 4:**
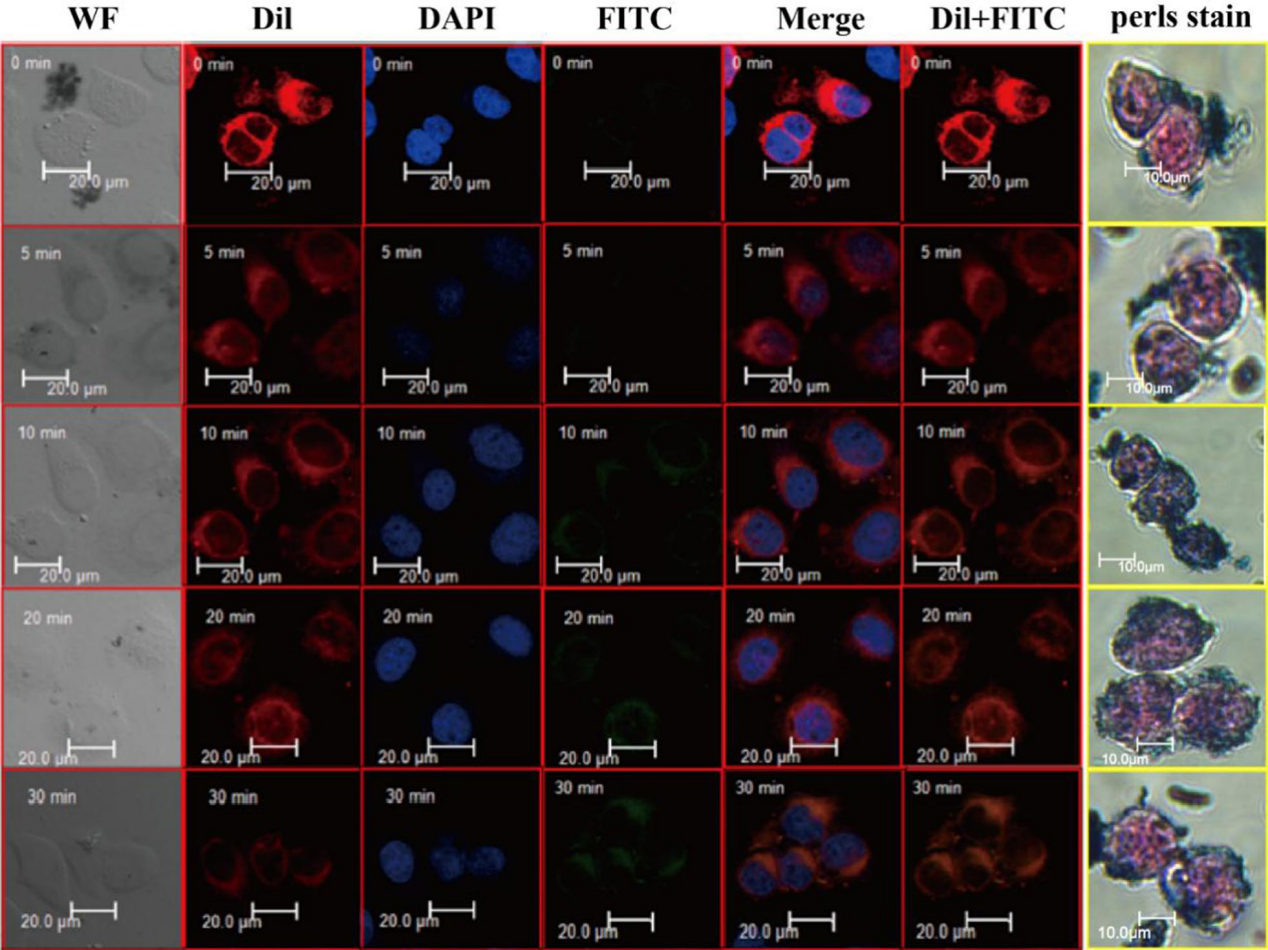
Study on the adsorption of GPC3-IML on the surface of HCC cells by laser confocal microscopy (LCM). First Column: Images in bright field; Second Column: Confocal images of cells showed the distribution of Dil-derived fluorescence (red); Third Column: Confocal images of cells showed nuclear staining with DAPI (blue); Fourth Column: Confocal images of cells showed the distribution of FITC-derived fluorescence (green); Fifth Column: Overlaid images; Sixth Column: Merge images of cells showed the distribution of Dil-derived fluorescence (red) and FITC-derived fluorescence (green); Seventh Column: Confocal images of cells showed the distribution of prussian blue staining

Membrane expression of GPC3, a specific marker of HCC, was designed as the target of HCC cells to determine the uptake efficiency of FITC labeled GPC3 immunoliposomes and to investigate the recognition behavior of tumor cell capture. Laser confocal microscope was used to observe the fixation of basal cells adhered on the slide of microscope (Fig. 4). The statistically analysis of fluorescence intensity values for confocal images in different groups were displayed in Additional file 3: Figure S3. When the sample containing mixed CTCs, immunoliposomes would automatically search for CTCs and adhere to the cell surface. The number of immunoliposomes increased and gradually gathered around the CTCs. After a while, the cell surface would be surrounded by enough magnetosomes, and the magnetosomes stopped adhering anymore. Red fluorescence indicated cell membrane dye Dil, which overlapped well with FITC labeled GPC3 immunoliposomes (green fluorescence in the figure), suggesting that the immunomagnetic lipid vesicles were distributed around the cell membrane. In addition, the prepared immunoliposomes showed good stability and can be used to capture tumor cells for at least 3 months after preparation.

Prussion Blue Staining results revealed that there was blue signal on the cell surface, indicating that GPC3-IML was absorbed by the cell and the material targeted. As time went on, the blue signal around the cell gradually increased. The binding of GPC3 on the surface of HCC cells reached the peak at 10 min.

### Clinical verification of HCC samples

EpCAM, Vimentin and GPC3 immunoliposomes were used for the CTCs capture in the blood of HCC patients. Meanwhile, the fluorescence microscope was employed to obtain the captured CTC counts and the capture efficiency of each magnetic particle, as shown in Table 1. The specific clinical characteristics of enrolled cases were shown in Table 2. Subsequent statistical analysis was performed for the clinical data of 21 patients with HCC. The results showed that the CTC counts captured by the immunoliposomes with specific marker GPC3 was similar to that obtained by EpCAM or Vimentin immunoliposomes (no statistical significance reached).

**Table 1:** Clinical characteristic of HCC patients and corresponding relationship with CTCs before treatment (*n* = 21)

Clinical characteristics	Average CTC numbers			Average CTC numbers			
	Total	EpCam	Vimentin	HSPG	HSPG/total HSPG serial (%)	GPC3	GPC3/total HSPG serial (%)
Age, years							
> 50	8	5	3	2	10.00	3	15.00
≤ 50	10	5	5	2	11.11	2	11.11
Gender							
Male	9	5	4	2	10.53	2	10.53
Female	10	6	3	2	11.11	2	11.11
Number of tumors							
Singleton	9	4	4	2	10.00	3	15.00
Multiple	9	5	4	2	9.09	4	18.18
AFP							
> 300 ng/mL	12	6	6	2	8.70	3	13.04
≤ 300 ng/mL	8	4	3	2	9.52	3	14.29
Tumor size (cm)							
> 5 cm	9	4	4	2	10.00	3	15.00
≤ 5 cm	9	5	4	2	10.00	3	15.00
BCLC stage							
0	7	4	3	2	15.38	0	0.00
A	8	5	3	2	10.00	3	15.00
B	8	3	5	3	11.11	5	18.52
C	12	6	6	2	9.09	3	13.64
PIVKA							
> 70 ng/ml	9	5	4	2	9.52	3	14.29
≤ 70 ng/ml	8	5	3	3	15.00	4	20.00
Large vessel tumor thrombus							
Yes	12	6	6	2	9.09	3	13.64
Non	8	4	3	2	9.52	3	14.29
MVI							
= M0	8	5	3	2	10.00	3	15.00
≠ M0	9	5	5	2	9.52	3	14.29

HSPG serial of immunoliposomes contain IMLs constructed with antibody of GPC1, GPC3, SDC1, SDC2, and common antibody of HSPG

*CTC* circulating tumor cell, *HSPG* heparan sulfate proteoglycans, *AFP* alpha-fetoprotein, *PIVKA* protein induced by vitamin K absence, *CEA* carcinoembryonic antigen

**Table 2:** Clinicopathological characteristics of HCC patients (*n* = 21)

Clinical Characteristics	Variables	Available data (n)	n (%)
Age, years	20–30/30–40/40–50/50–60/60–70/ > 70	21	2(9.52%)/2(9.52%)/7(33.3%)/5(23.8%)/4(19.0%)/5(23.8%)
Gender	Male/Female	21	18(85.7%)/3(14.3%)
TNM	T1bN0M0/T2N0M0/T4N0M0/T4N1M1	11	6(54.5%)/1(9.0%)/3(27.3%)/1(9.0%)
BCLC	0/A/B/C	14	1(7.1%)/7(50.0%)/2(14.3%)/4(28.6%)
Immunohistochemistry	GPC3(+)/ GPC3(−)	13	7(53.8%)/6(46.2%)
	Hep-1(+)/ Hep-1(−)/None	18	11(61.1%)/1(5.5%)/6(33.3%)
	CD34(+)/CD34(−)/None	18	10(55.6%)/2(11.1%)/6(33.3%)
	HBsAg(+)/HBsAg(−)	18	14(77.8%)/4(22.2%)
	Arginase(+)/Arginase(−)/None	18	8(44.4%)/1(5.5%)/9(50.0%)
	AMACR(+)/AMACR(−)/None	18	8(44.4%)/5(27.8%)/5(27.8%)
	CK7(+)/CK7(−)	18	1(5.5%)/17(94.4%)
	GS(+)/GS(−)	18	2(11.1%)/16(88.9%)
	HSP70(+)/HSP70(−)	18	11(61.1%)/7(38.9%)
	CD10(+)/CD10(−)	18	2(11.1%)/16(88.9%)
	CK19(+)/CK19(−)	18	1(5.5%)/17(94.4%)
	CD31(+)/None	18	9(50.0%)/9(50.0%)

**Fig. 5:**
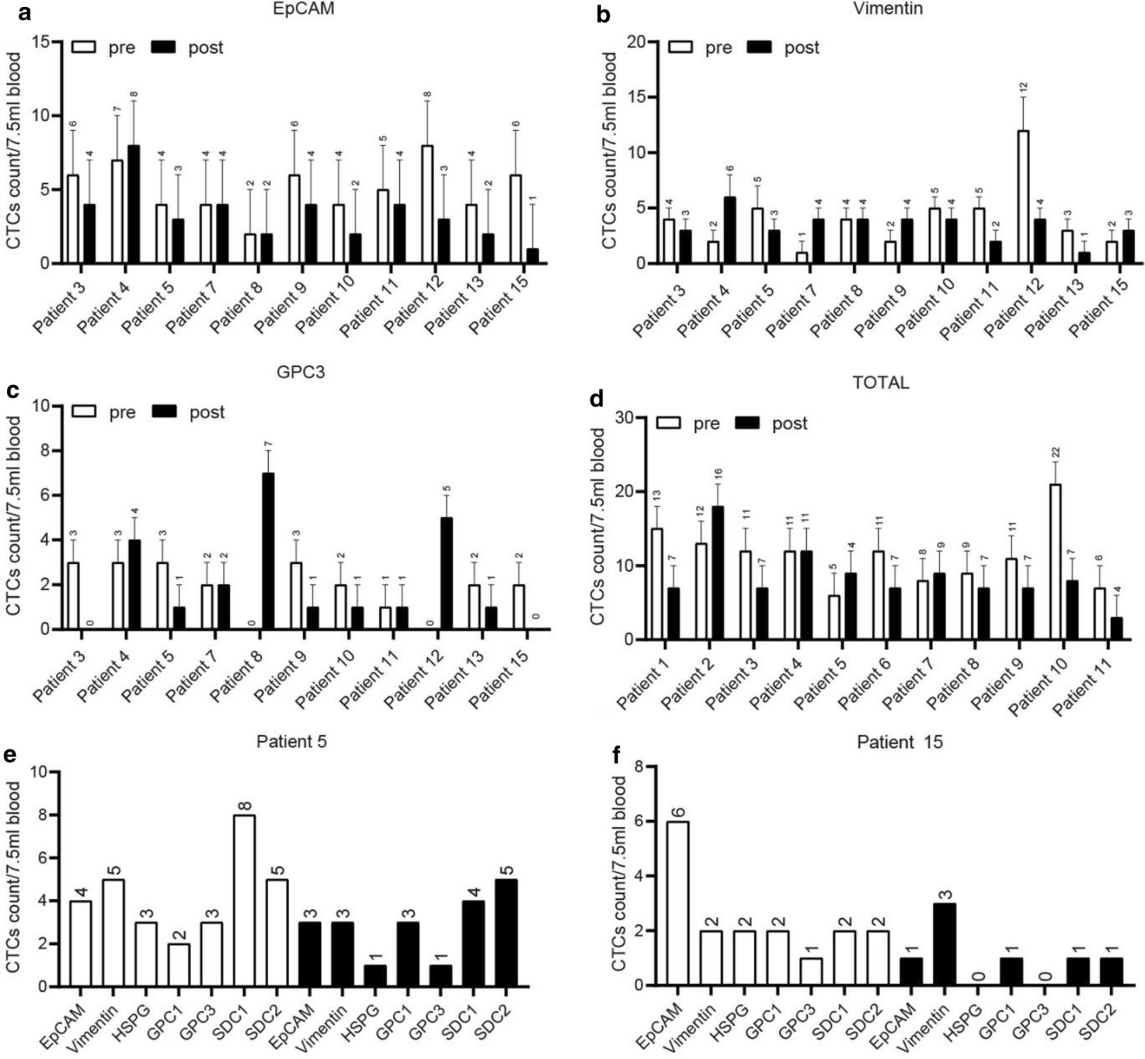
Clinical verification of HCC samples. Statistical analysis between CTCs of HCC patients and EpCAM-IML (**a**), Vimentin-IML (**b**) and GPC3-IML (**c**) focusing on the time that before and after the operation. **d** The total captured numbers of cells by IMLs were analyzed on the time that before and after the operation. The captured CTC of patient No. 5 (**e**) and patient No. 15 (**f**) by the seven immunoliposomes before and after treatment

Further analysis was conducted focusing on the quantity and proportion of the three immunoliposomes capturing CTCs of HCC patients under different age, gender, tumor number, tumor size, AFP, BCLC stage, and PIVKA. The results were shown in the Fig. 5a–d. According to the results, there was a generally decreased total count of CTCs detected by the three markers 1 week after the surgery for HCC, and especially the count of CTCs captured by EpCAM immunoliposomes before treatment was higher than that after treatment. However, some opposite cases of elevated count number  after surgery were found in the count of CTCs captured by GPC3 immunoliposomes. Further comparison was made in terms of the count of CTCs captured by more immunoliposomes before and after R0 resection in two patients with HCC (Fig. 5e, f). The results of one patient showed an unchanged patern of counting distribution of 7 IMLs, and the results of the other presented a decreased count except for the count of CTC captured by GPC3.

### Correlation of GPC3-IML with clinical index

**Fig. 6:**
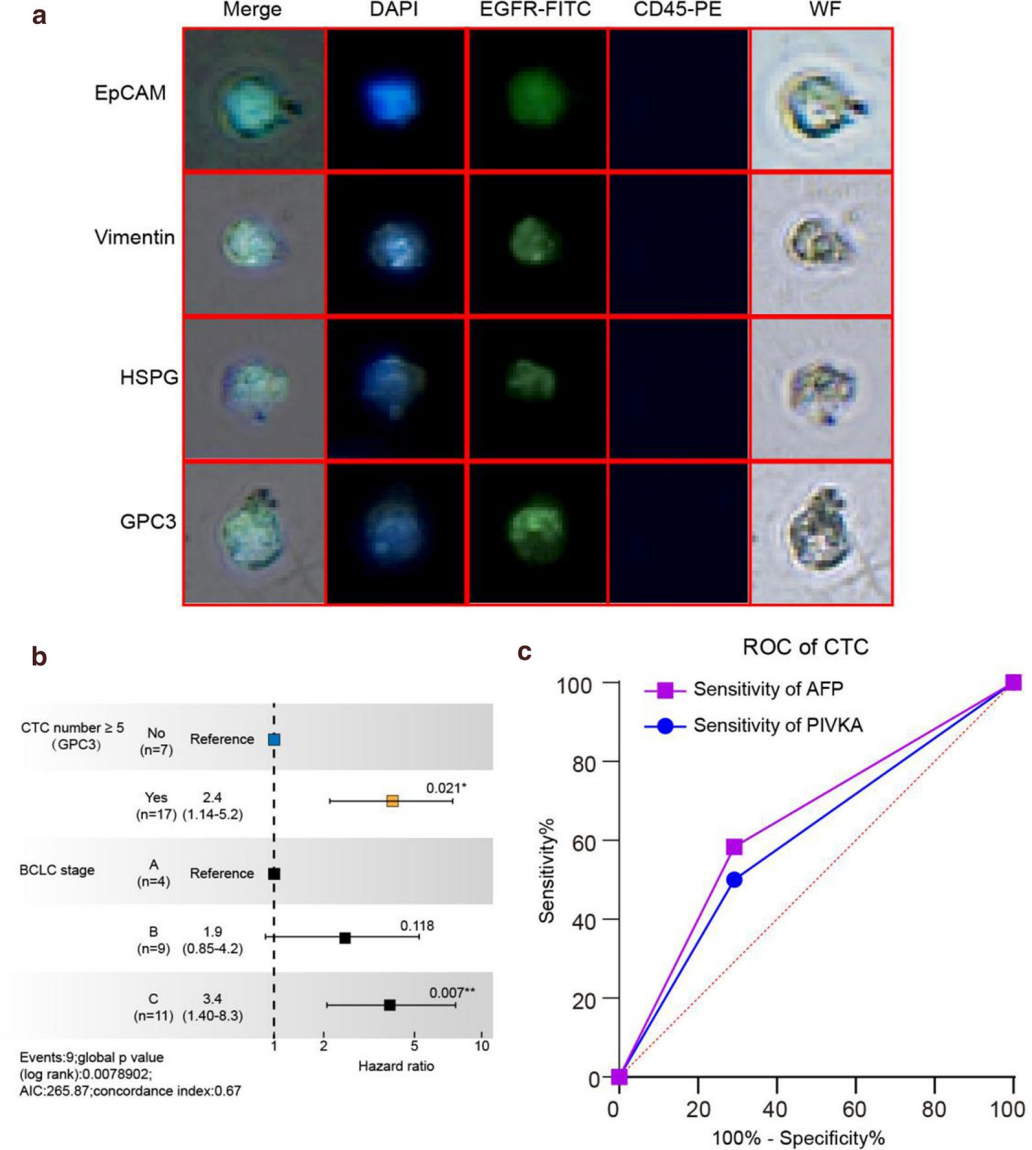
Correlation of GPC3-IML with clinical index. **a** The results of GPC3-IML separating tumor cells in the peripheral blood of patients with HCC; **b** Correlation between the number of captured CTC and clinical indexes; **c** the sensitivity and specificity of CTC count and AFP by ROC curve

The results of GPC3-IML separating tumor cells in the peripheral blood of patients with HCC were shown in the Fig. 6a. The results of immunofluorescence staining showed that the tumor cells separated from the blood sample of patients with HCC were similar in shape and size, with a quite consistent staining state as well. In this regard, the seven immunoliposomes constructed in this study can effectively separate tumor cells from peripheral blood of patients with HCC, and can detect the presence of CTCs.

**Fig. 7:**
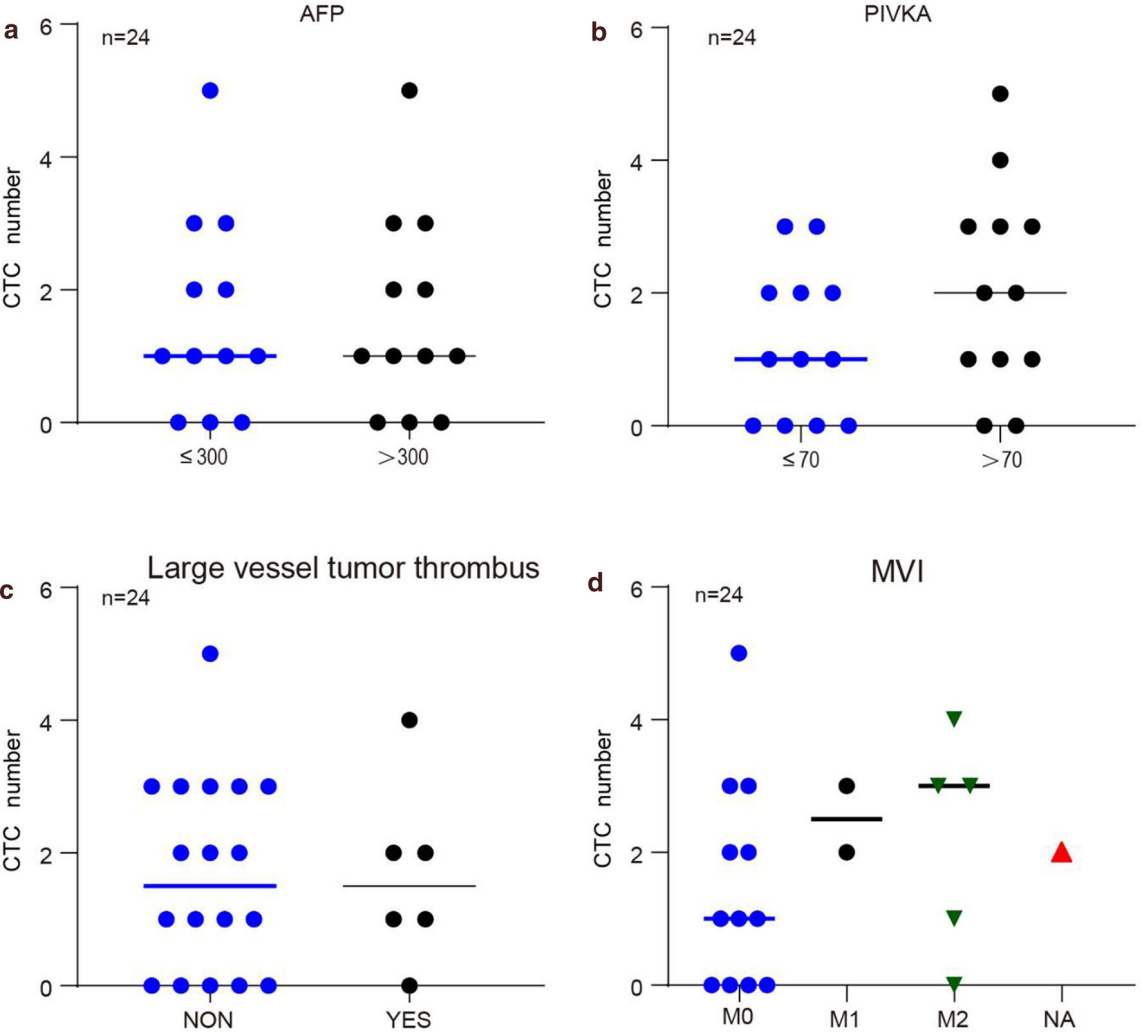
The count of CTCs were compared among patients grouped by AFP (**a**), PIVKA (**b**), microvascular tumor thrombus (**c**) and MVI (**d**)

In our previous preliminary study of 24 patients, "positive" CTC counts (≥ 5 PV-CTC per 7.5 ml of blood) were associated with BCLC (P = 0.055). By using this cut-off value, the positive result was significantly correlated with stage C [Log-Rank test with P = 0.019, hazard ratio (HR) = 2.28, Fig. 6b], which was an independent predictor in multivariate analysis after adjusting tumor stage [P = 0.021; HR = 2.4, 95% confidence interval (CI) = 1.14–5.2]. Meanwhile, ROC curve (Fig. 6c) indicated that the sensitivity and specificity of CTC count and AFP were higher than that of PIVKA. However, there was a limited correlation of CTC count with AFP and PIVKA (sensitivity = 31.7%, specificity = 84.9). Furthermore, the count of CTCs were compared among patients grouped by AFP, PIVKA, microvascular tumor thrombus and MVI, and the results showed that there was no correlation in the count distribution (Fig. 7a–d).

### NGS-based detection of mutation abundance and consistency

**Fig. 8:**
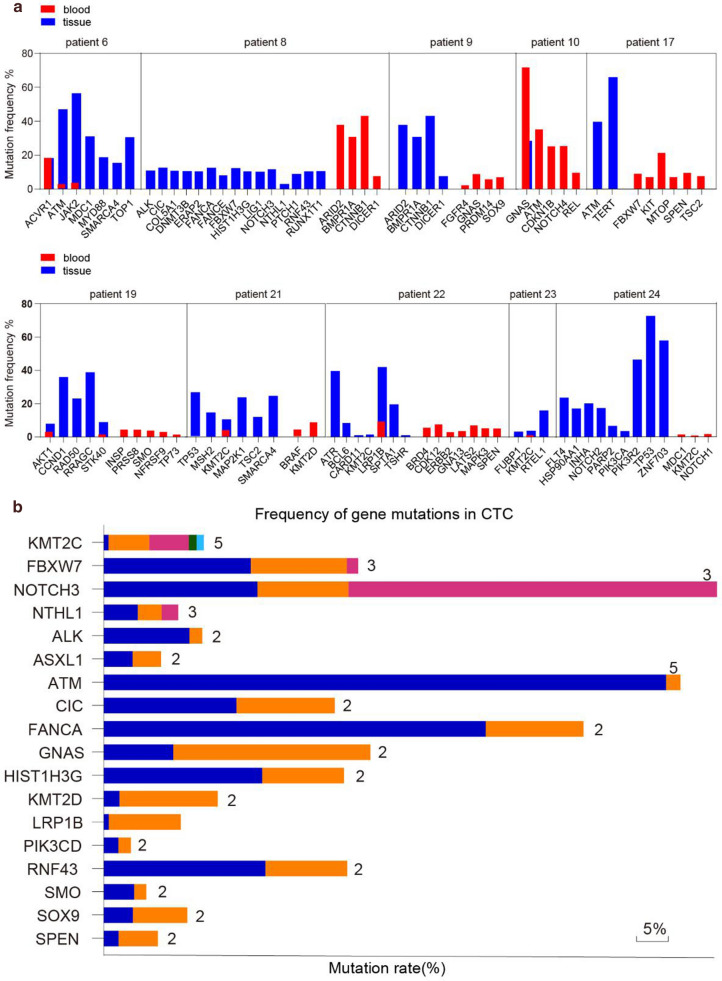
Detected mutations in CTCs. **a** The consistency between CTC-NGS and tissue-NGS; **b** The top18 genes list of CTC-NGS mutation

In our experiment, the changes of major oncogenes/tumor suppressor genes were searched in HCC, followed by the analysis of gene mutations based on high-throughput sequencing of CTCs and HCC tissues. The results of 24 cases of NGS detection based on CTC-DNA were analyzed, and 10 of them were selected for the corresponding tissue-NGS detection. The results showed that the consistency between CTC-NGS and tissue-NGS was 60% (Fig. 8a). The top18 gene lists of CTC-NGS mutation were shown in Fig. 8b. It was worth noting that KMT2C gene also showed high-frequency mutation in tissue-NGS detection results.

## Discussion

HCC is a highly heterogeneous disease, whether clinically or at the molecular level. So far, conventional diagnostic methods have not been able to identify HCC patients with a high risk of metastasis and whose tumor cells escape or invade the peripheral blood during surgery [24]. The measurement of CTCs in peripheral blood can be an alternative diagnostic test, which can be regarded as a "liquid biopsy" and provide real-time information of patients' current disease status [25, 26]. Previous studies have proofed that CTC count can provide valuable information for prognosis analysis. However, through gene molecular analysis of CTCs, combined clinical diagnosis can provide an updated understanding of the dynamic changes of tumor occurrence and development, and thus broaden the molecular mechanism of HCC, a fatal malignant tumor [27–29]. In the era of cancer precision medicine, there is a high demand for precise biomarkers, which can be used in a minimally invasive manner before, during and after treatment [30–32].

According to the prior research, GPC-3 is significantly upregulated in human HCC, exhibiting an intimate association with the occurrence and development of HCC. It is considered as a potential therapeutic target of HCC, and its antibody possesses an antitumor effect [33]. This study constructed an optimized platform based on GPC-3 (a subtype of HSPG) to detect CTCs in HCC. Our platform was proven to have high specificity and sensitivity for the detection of rare CTCs in the blood sample of HCC patients, and further comparison was carried out with the separation ability of EpCAM and Vimentin, which are universal types of pan-cancer. As far as we know, it is not only the first time to establish HSPG/GPC-3 immunoliposomes for HCC, but also to realize the dynamic detection of CTC separation, capture and counting in HCC patients before and after operation. Our study provides a clinical evaluation about how the HSPG/GPC-3 immunoliposomes in the change of captured CTC count related with clinical indicators in HCC patients? Corresponding results revealed that there was a correlation between the "positive" CTC counts (≥ 5 PV-CTC per 7.5 ml of blood) and BCLC (P = 0.055). In this regard, dynamic tracking and detection of CTCs and their molecular level changes in HCC patients may be a novel real-time indicator to reflect the risk of recurrence in HCC patients. It may in turn contribute to supplying a therapeutic window or an indicator of the risk of disease progression, thus providing convenience and increasing safety.

At present, CTC detection methods including CellSearch system have been utilized in several types of metastatic cancer to explore the value of CTC count in prognosis research [34–37]. However, the CellSearch system is unable to detect the decrease in CTC count due to EpCAM downregulation caused by EMT. Accordingly, a GPC-3-based highly efficient-cell enrichment, separation, identification and detection system for CTCs of HCC was established by our group in this study. The microstructure of immunoliposomes was determined by AFM. It can be clearly observed that one or more magnetic particles of 10–100 nm in diameter were wrapped in antibody-bound immunomagnetic lipid vesicles with a diameter of 150–700 nm. MTT results revealed that GPC-3 immunoliposomes had lower toxicity and higher biocompatibility, which laid a foundation for clinical separation of CTCs and the subsequent research. Meanwhile, under confocal microscope, the interaction between immunoliposomes and HCC cells indicated high affinity and strong interaction with tumor cells.

KMT2C (also known as MLL3) is a member of the mixed-lineage leukemia (MLL) family, which comprises of KMT2A, MKT2B, KMT2D, KMY2F and KMT2G [38]. In the recent decades, duing to the progression in high-throughput sequencing and other research methods, KMT2C has been increasingly demonstrated to play an important role in many solid tumors. Mutations of KMT2C (MLL3) have been found in pancreatic cancer, gastric cancer, ovarian cancer, colorectal cancer, cholangiocarcinoma, bladder transitional cell carcinoma, etc. [39–42]. A previous genomics study showed that 47% of gastric cancer patients had mutations in KMT2C or KMT2D [43, 44]. In this study, 24 cases of CTC (DNA)-NGS data were collected, with the collection of tissue-NGS data in the enrolled 10 cases. Our study reported a 60% consistency between CTC-NGS and tissue-NGS, among which, KMT2C gene exhibited the highest mutation frequency from both CTC and tissue. The consistency between DNA from tissue and blood was also reported in the past [45]. Without doubt, it is necessary to examine these important findings on the basis of larger sample size, for example, by investigating whether mutations in normal tissue are associated with an increased risk of local disease recurrence. Hence, our research group will carry out further analysis on the molecular mechanism of downstream genes on a larger scale, aiming at exploring molecular markers for intra-tumor and inter-tumor heterogeneity and corresponding functional verification, so as to provide abundant useful data for molecular therapy.

## Conclusion

To sum up, according to a comprehensive analysis in our study, the established enrichment and separation detection system for CTCs of HCC can effectively achieve real-time dynamic monitoring of CTCs in patients with HCC. The development of NGS technique creates a new approach for the analysis of molecular defects in HCC samples. DNA from tissue and peripheral blood were used in our study to detect the abundance consistency of mutant genes, with the purpose to reveal the main cancer-driving genes in HCC, combined with in-depth analysis of its related carcinogenic pathway. There have been some studies emphasizing on the integration of ctDNA with imaging results (such as RECIST standard or the sum of target lesions). However, blankness still exists in comprehensive study on clarifying the correlation with clinical detection indicators on the basis of dynamic data of CTCs from peripheral blood. It is expected to provide important clinical reference for early diagnosis, preoperative and postoperative analysis and treatment effect evaluation of HCC.

## Materials and methods

### Experimental materials and equipment

HCC patients, in Shanghai Eastern Hepatobiliary Surgery Hospital, with imaging diagnosis confirmed were studied. This study was approved by the ethics committee of Shanghai Eastern Hepatobiliary Surgery Hospital (approval code: EHBHKY2020-k-024), these samples were collected from May 2019 to January 2020 and all enrolled patients signed the informed consent. Eligibility criteria: (1) patients with imaging diagnosis confirmed by Hepatobiliary Multidisciplinary Team (MDT) Cooperation Group discussion, with no previous anti-tumor treatment history such as surgery, radiotherapy or chemotherapy, and with age ≥ 18 years; (2) patients with measurable or evaluative tumor lesions displayed by CT or MRI (according to RECIST 1.1 criteria), and with KPS score > 60 points; (3) patients with good blood function: hemoglobin ≥ 8 g/dL, absolute neutrophil count ≥ 1.5 × 10^9^/L, and platelet count ≥ 100 × 10^9^/L; (4) patients without heart failure, uncontrollable chest pain, myocardial infarction or cerebral infarction within 12 months before the study.

Sample collection and processing: (1) Sample processing: A sample of 7.5 mL peripheral blood was collected from each HCC patient using medical anticoagulant blood collection tube, and the anticoagulant was EDTA▪K2. The collected blood was stored at 4℃, and should be tested within 72 h. (2) Testing indexes: (1) CTC counting in peripheral blood of patients with HCC (immunomagnetic separation and immunofluorescence identification for counting); (2) detection of gene mutation in CTCs of HCC (panel sequencing); (3) CTC detection of genes in HCC. 3) Clinical information collection: clinical information of patients mainly included: name (sample No.), gender, age, clinical stage, pathological classification, and medication, etc.

HCC cell lines MHCC97-L and Huh-7 (American Type Culture Collection [ATCC]); DMEM culture medium, fetal bovine serum and trypsin (Gibco); EpCAM, HSPG, Vimentin and GPC3 monoclonal antibodies (Abcam); DAPI staining solution (Sigma); CD45-PE (eBioscience); EpCAM antibody derivatives and magnetic nanoparticles (Huzhou Lieyuan Medical Laboratory Co., Ltd.); Distearoyl phosphatidylethanolamine-polyethylene glycol (DSAPC-APCG; Avanti); cholesterol (Chol), dichloromethane and other common reagents (Chinese Medicine Co., Ltd.); fluorescence microscope (OLYMPUS B × 61; Olympus Corp, Japan); and flow cytometer (BD FACS caliber; Becton Dickinson, USA).

### Preparation of GPC3 immunoliposomes (GPC3-IML)

Preparation of GPC3-IML was realized by reverse phase evaporation method. An amount of 5 mg DOPC and 5 mg Chol were weighed and added to two 50 ml three-neck flasks. Then, 1.0 ml of Fe_3_O_4_-HMN solution was weighed and dissolved in 3.0 ml of CH_2_Cl_2_ after the removal of ethanol. The obtained Fe_3_O_4_-HMN/CH_2_Cl_2_ was transferred to the above three-neck flasks. The round bottom flask was emulsified for 6 min with the probe-type ultrasonic instrument under the condition of ice bath. Simultaneously, 2 mg GPC3-GHDC was dissolved in 6 ml of double distilled water (ddH_2_O) and slowly added into the three-neck flasks. At the end of ultrasound, CH_2_Cl_2_ was removed by rotary vaporizer, and the IMLs were obtained after magnetic separation solution washed three times. Meanwhile, EpCAM and Vimentin antibody modified IML were obtained through the same operation procedure. The preparation process of magnetic beads was as followed in Additional file 4: Figure S4.

### Characterization of GPC3-IML

Particle size and potential of IML were measured by Zetasizer Nano-ZS 90 (Malvern instruments Ltd., UK). Atomic force microscopy (AFM) was used to observe the morphology of different IMLs. Hysteresis loop of magnetic particles was detected by using PPMS-9 (QUANTUM DESIGN, USA). UV spectrophotometer was applied to scan the UV absorption peak of IML solution. In the next step, our experiment focused on further confirmation of the existence of antibody on the surface of IMLs and qualitative analysis of antibody content. To be specific, BCA protein quantitative analysis was performed to make quantitative analysis of the antibody content on the surface of IMLs. Meanwhile, polyacrylamide gel electrophoresis (PAGE) was used to detect the antibody content on the surface of IMLs and to confirm the presence of antibodies.

### Capture efficiency analysis of IML with different antibodies

The MHCC97-L and Huh-7 cells were placed in PBS after counting, and 50 μL IML was added and mixed for cell capture experiments. FITC-labeled anti-CK19 antibody (antiCK19-FITC) staining was then performed, and the captured cells were counted under a fluorescence microscope. In particular, 10 μL anti-CK19-FITC was added to the cells after magnetic separation and stored away from light for 15 min at room temperature. Each sample was then placed in a magnetic separation frame for 2 min and washed 3 times with PBS buffer for 2 min each time. The capture efficiency of EpCAM-IML, Vimentin-IML and GPC3-IML among different cells were determined using the above methods. The substrates contained PBS buffer, whole blood or lysed blood. The number of fluorescence labeled cells was analyzed by flow cytometry.

### Separation of HCC cells by GPC3-CTC system

MTT method was used for toxicity analysis of GPC3-IML. As for the specific procedures, the single-cell suspension was prepared after trypsin digestion of HCC cells, followed by the addition of serum-containing medium to neutralize the trypsin. Cell concentration was calculated by cell counting plate after cell dilution. After that, the treated HCC cells were inoculated into the 96-well plate, with 8,000 cells inoculated into each well, and the medium was 100 μl per well. After overnight culture, IML at gradient concentration was added to cells to adjust the final concentration to 0, 10, 50, 100, 200, 500, and 1000 μg/ml, respectively. After incubation at 37℃ for 48 h, 10 μl of 5 mg/mL MTT reagent was added into each well, followed by another incubation for 3 h with the observation of obvious formazan crystal formation under the microscope. After the removal of the medium, 150 μL DMSO solution was added into each well to dissolve the crystal. The results of the experiment were collected at wavelength of 490 nm by using multi-functional Microplate Reader SpectraMaxM5/M5e (MolecularDevices) for data analysis and processing. Prussion Blue Staining adopted nuclear fast red by using the Prussion Blue Staining Kit (Art. No.: BB-44371).

### Classification and counting of CTCs

Similar to the classic CellSearch system, the separation and identification scheme of circulating tumor single cells of HCC patients were as follows: The main components were CTC IML separation reagent and CTC immunofluorescence identification reagent, including anti-HSPG and anti-GPC3 IMLs, DAPI fluorescent dye for nuclear staining, EGFR-FITC and SYN antibodies (SYN-PE) for tumor marker identification and CD45-PE reagent for leukocyte identification.

A sample of 7.5 ml of peripheral blood was taken from each patient at the time point of test, and then put into EDTA anticoagulant tube. After shaking, it was stored in 4 °C refrigerator and transported to the laboratory of CTCs Test Center within 24 h. In this study, the CTCs in the peripheral blood were enriched and screened by the self-made immunomagnetic spheres. The whole cells with nuclei were identified by DAPI, and then the epithelial cells were distinguished from the leukocytes by fluorescent-dye-labeled monoclonal antibody CD45 and CK19 staining. The evaluation criteria of CTCs should be identified and be qualified with FITC + ,CK19 + , DAPI + , and CD45-. After that, CTCs were counted by the polychromatic fluorescent Cell Counter.

### NGS analysis of captured HCC cells by GPC3-IML

This experiment was based on the sequencing by ligation using four fluorescent-dye-labeled oligonucleotides. Prior to sequencing, the DNA template was amplified by emulsion PCR, and the 3′-end modified particles should be deposited on the slides. The substrate used for sequencing by ligation was a mixture of 8 base fluorescent probes. The DNA sample can be labeled by the probe according to the location of the sequence. DNA ligase preferentially ligated probes matched with the template, and triggered the generation of fluorescence signals at this site.

### Statistical analysis

Data of each group was processed by SPSS 19.0 statistical software. The measurement data was expressed mean ± SD, with α = 0.05 as the inspection level (two-tailed), and the rank-sum test was used for statistical analysis. The diagnostic sensitivity, specificity, validity, positive predictive value and negative predictive value were calculated at the same time. *P* < 0.05 meant that the difference was statistically significant.

## Supplementary Information

**Additional file 1: Figure S1.** The TEM images of GPC3-IML, EpCAM-IML and Vimentin-IML.

**Additional file 2: Figure S2.** Study on Cytotoxicity of GPC3-IML. The cell viability of GPC3-IML in MHCC97-L cell line (**A**), Huh-7 cell line (**B**) and WRL-68 cell line (**C**, Negative Control) under the cell concentration: 0 μg/mL, 10 μg/mL, 50 μg/mL, 100 μg/mL, 200 μg/mL, 500 μg/mL and 1000 μg/mL, time point at 0 h, 0.5 h, 2 h and 24 h respectively. **D** The cell viability under the cell concentration of 200 μg/mL by MTT assay.

**Additional file 3: Figure S3.** The statistically analysis of fluorescence intensity values for confocal images.

**Additional file 4:** **Figure S4.** The scheme of magnetic beads preparation process.

## Data Availability

All data generated or analysed during this study are included in this published article [and its additional information files].
