# Correction to: The clinical significance of CTC enrichment by GPC3-IML and its genetic analysis in hepatocellular carcinoma

**DOI:** 10.1186/s12951-021-00889-2

**Published:** 2021-06-12

**Authors:** Bin Yi, Tian Wu, Nan Zhu, Yao Huang, Xiaoyu Yang, Lei Yuan, Yingjun Wu, Xiaofei Liang, Xiaoqing Jiang

**Affiliations:** 1 Department of Organ Transplantation, Eastern Hepatobiliary Surgery Hospital, Second Military Medical University, Shanghai, China; 2 Jukang (Shanghai) Biotechnology Co. Ltd, 28, Xiangle Rd, Shanghai, 201800 China; 3 Department I of Biliary Tract, Eastern Hepatobiliary Surgery Hospital, Second Military Medical University, No. 225, Changhai Rd, Shanghai, 200438 China

## Correction to: J Nanobiotechnol (2021) 19:74 10.1186/s12951-021-00818-3

Following publication of the original article [1], the authors identified that there was a problem in Fig. 4 when they reviewed the data of article.

**Fig. 4:**
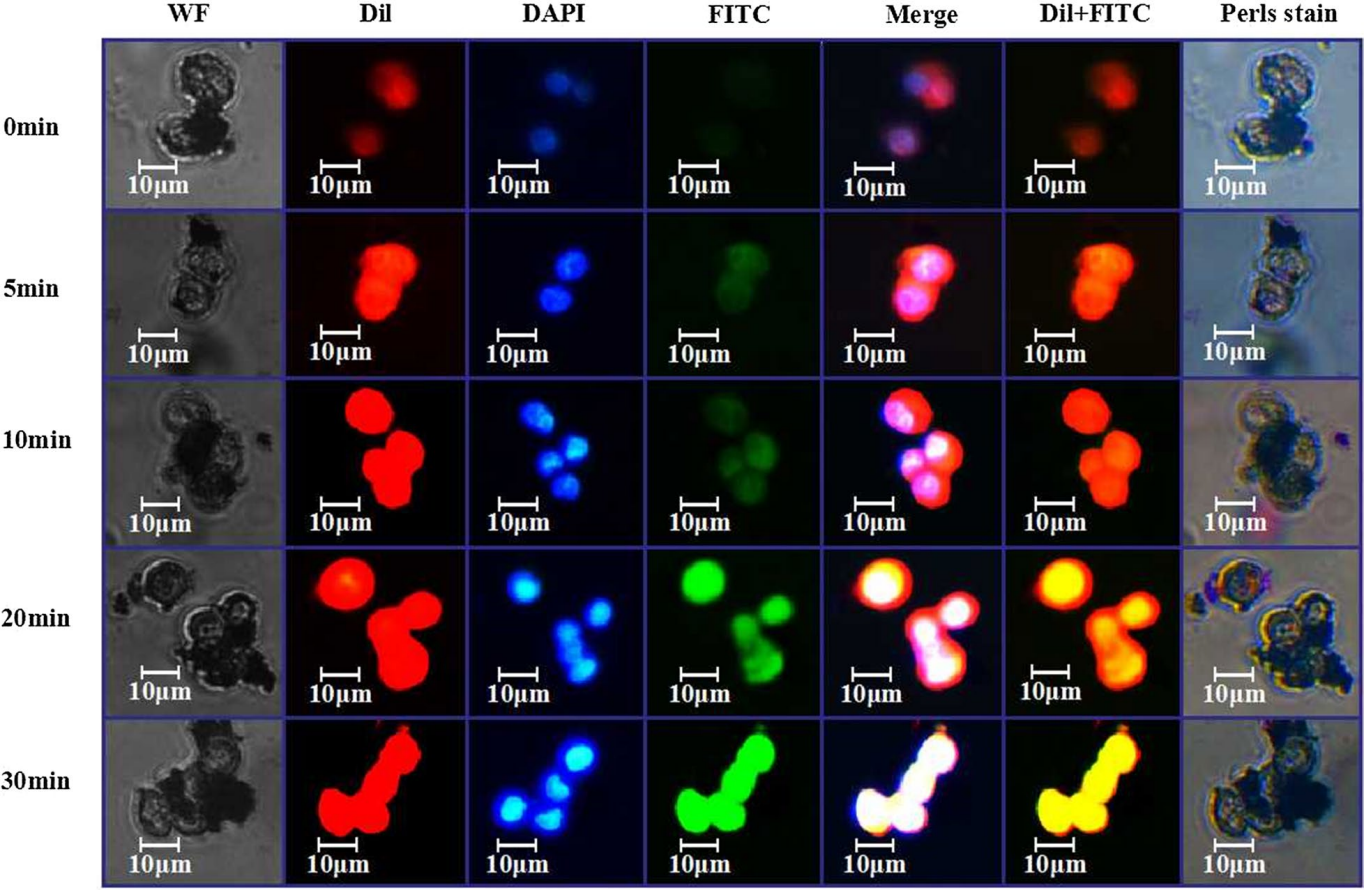
Study on the adsorption of GPC3-IML on the surface of HCC cells by fluorescence microscope. First Column: Images in bright field; Second Column: Images of cells showed the distribution of Dil-derived fluorescence (red); Third Column: Images of cells showed nuclear staining with DAPI (blue); Fourth Column: Images of cells showed the distribution of FITC-derived fluorescence (green); Fifth Column: Merged images; Sixth Column: Overlaid images of cells that showed the distribution of Dil-derived fluorescence (red) and FITC-derived fluorescence (green); Seventh Column: Images of cells showed the distribution of Prussian blue staining

In Fig. 4, the experiment of the Perls Stain in column 7 was not designed at the very beginning of the initial experiments. The aim of Perls Stain experiment is to show iron-containing in GPC3-IML. After the completion of simultaneous experiments of columns 1–6, we supplemented the experiment of Perls Stain by selecting the same cell line and performing at the corresponding time to prove this point. Thus, the image of Perls stain in column 7 is not consistent with the other columns in terms of cell number, magnification and locations, and is with wrong scale bar. Although the images of experiment of column 7 could prove the existence of GPC3-IML, the integrity and logic of the whole experiments were compromised. After the manuscript was accepted, we repeated the all experiments involved in each column of Fig. 4 in the same period in order to better express the significance of this study and to be better understood by readers.

The corrected Fig. 4 and the corrected figure caption are given below. The correction of these figures does not affect the results and conclusion. All authors agree to these corrections and apologize for these errors.

The correct Fig. 4 is published in this Correction article, and the original article has been corrected.

Corrected Fig. 4:

Attached instructions of **Experimental Procedure of Perls Staining for GPC3-IML:**

1. 1 × 10^4^ MHCC97-L cells were inoculated and 3 mL cell culture solution was added in a culture. The cells were cultured at 37 ℃ in a 5% CO_2_ incubator for 24 h.
2. After the change of culture solution, 20 μL GFAP-IML, 100 μL DAPI, 100 μL Dil and 50 μL GFAP-FITC were added into per culture plate.
3. The culture plate was fixed to the microscope and cell images was captured at 0 min.
4. The culture plate was placed in an incubator at a constant temperature, and the culture solution was drawn from the plate into the Eppendorf tube after 5 min. A very small amount of culture solution was added to take images.
5. The culture plate was still attached to the microscope and the culture solution was poured from the Eppendorf tube into the culture plate. 5 min later, the culture solution was drawn from the plate into the Eppendorf tube. A very small amount of culture solution was added to take images at 10 min.
6. The culture solution was poured from the Eppendorf tube into the culture plate and the plate was placed in an incubator at a constant temperature. After 10 min, the culture solution was drawn from the plate into the Eppendorf tube. A very small amount of culture solution was added to take images at 20 min.
7. The culture plate was still attached to the microscope and the culture solution was poured from the Eppendorf tube into the culture plate. After 10 min, the culture solution was drawn from the plate into the Eppendorf tube. A very small amount of culture solution was added to take images at 30 min.
