# Supplementary figures and images for: The clinical significance of CTC enrichment by GPC3-IML and its genetic analysis in hepatocellular carcinoma
Source: J Nanobiotechnology. 2021 Mar 16;19:74. doi: 10.1186/s12951-021-00818-3 (PMC7962223; doi:10.1186/s12951-021-00818-3)

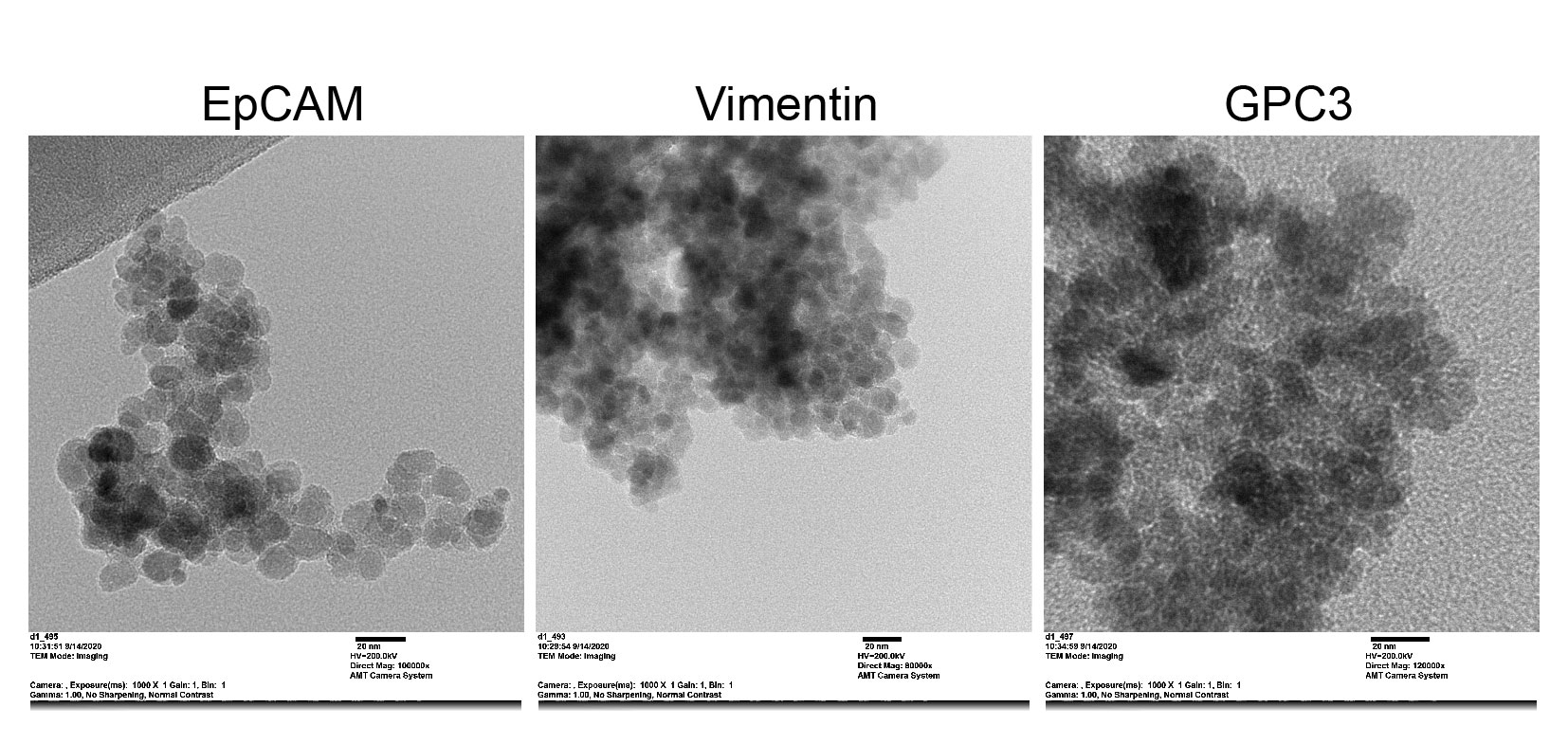

Supplement: Supplementary file 1 — Additional file 1: Figure S1. The TEM images of GPC3-IML, EpCAM-IML and Vimentin-IML. [file 12951_2021_818_MOESM1_ESM.jpg]

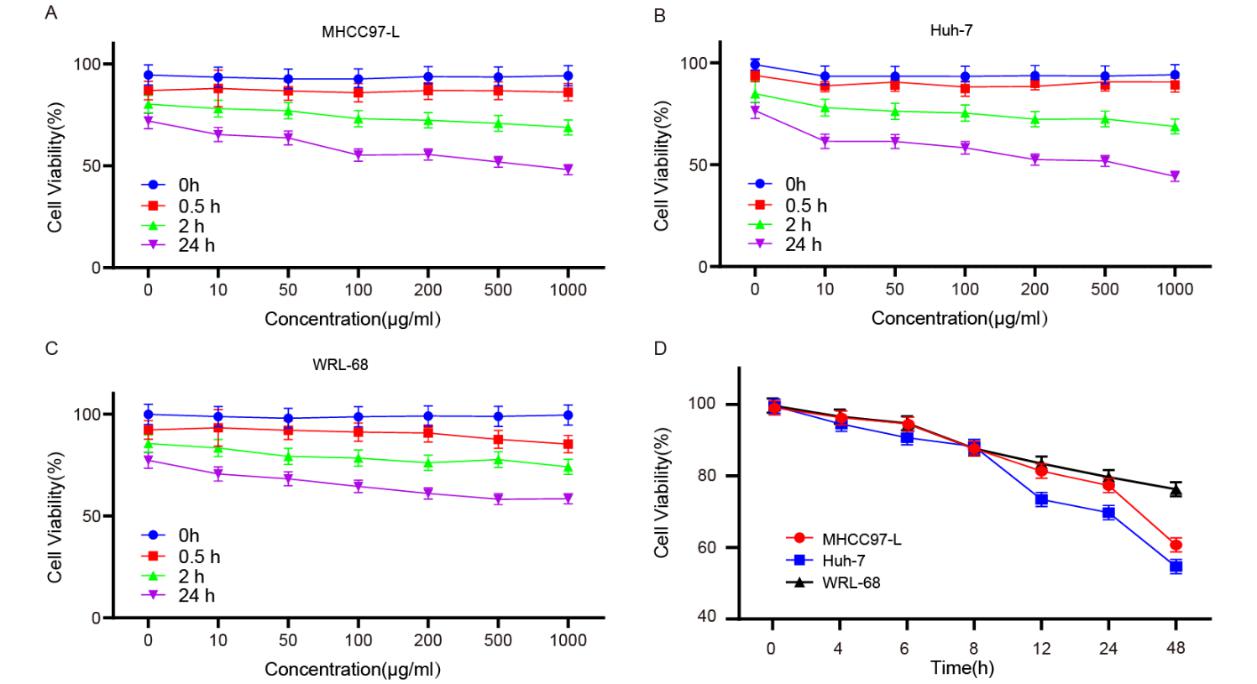

Supplement: Supplementary file 2 — Additional file 2: Figure S2. Study on Cytotoxicity of GPC3-IML. The cell viability of GPC3-IML in MHCC97-L cell line (A), Huh-7 cell line (B) and WRL-68 cell line (C, Negative Control) under the cell concentration: 0 μg/mL, 10 μg/mL, 50 μg/mL, 100 μg/mL, 200 μg/mL, 500 μg/mL and 1000 μg/mL, time point at 0 h, 0.5 h, 2 h and 24 h respectively. D The cell viability under the cell concentration of 200 μg/mL by MTT assay. [file 12951_2021_818_MOESM2_ESM.jpg]

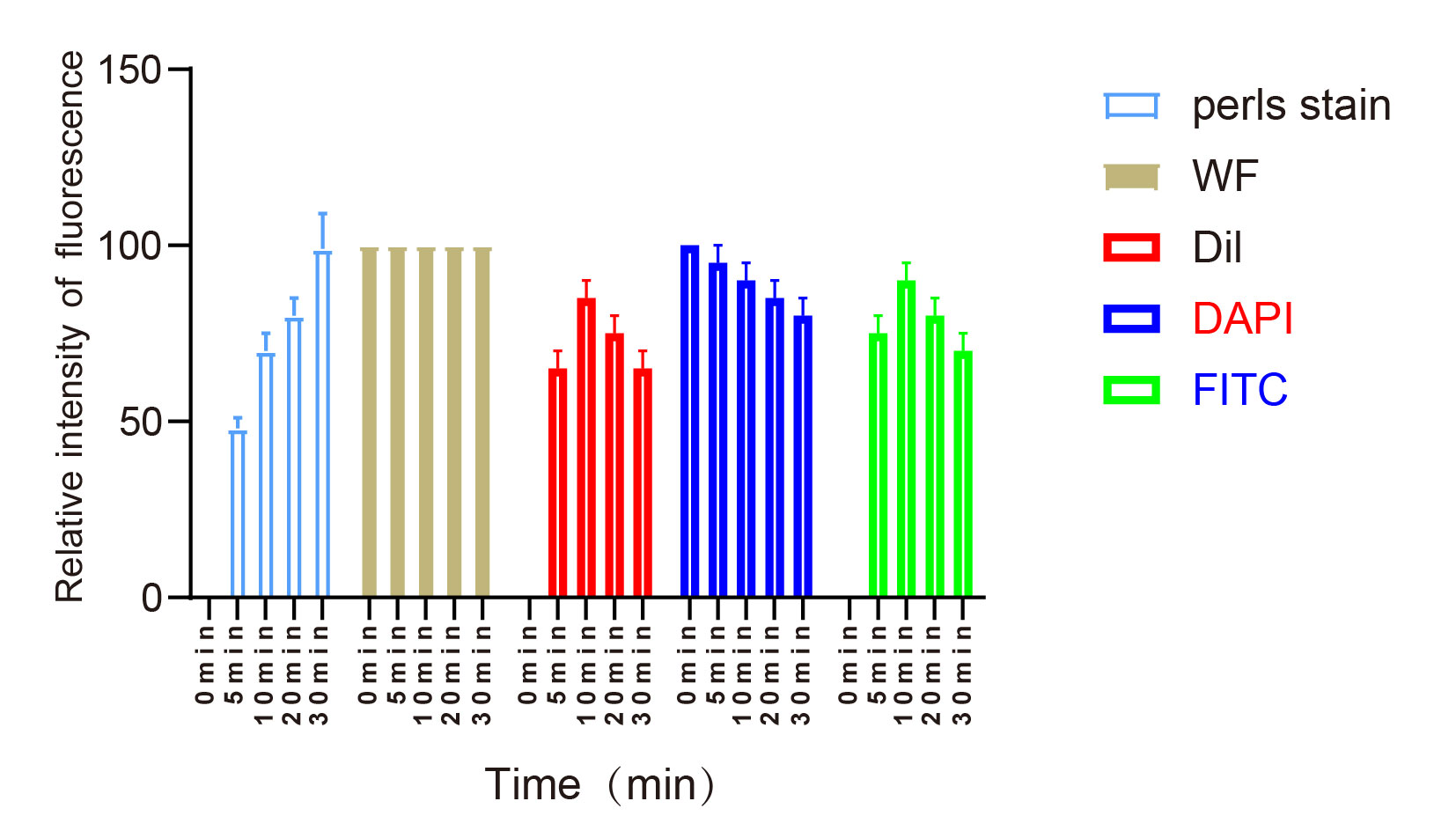

Supplement: Supplementary file 3 — Additional file 3: Figure S3. The statistically analysis of fluorescence intensity values for confocal images. [file 12951_2021_818_MOESM3_ESM.jpg]

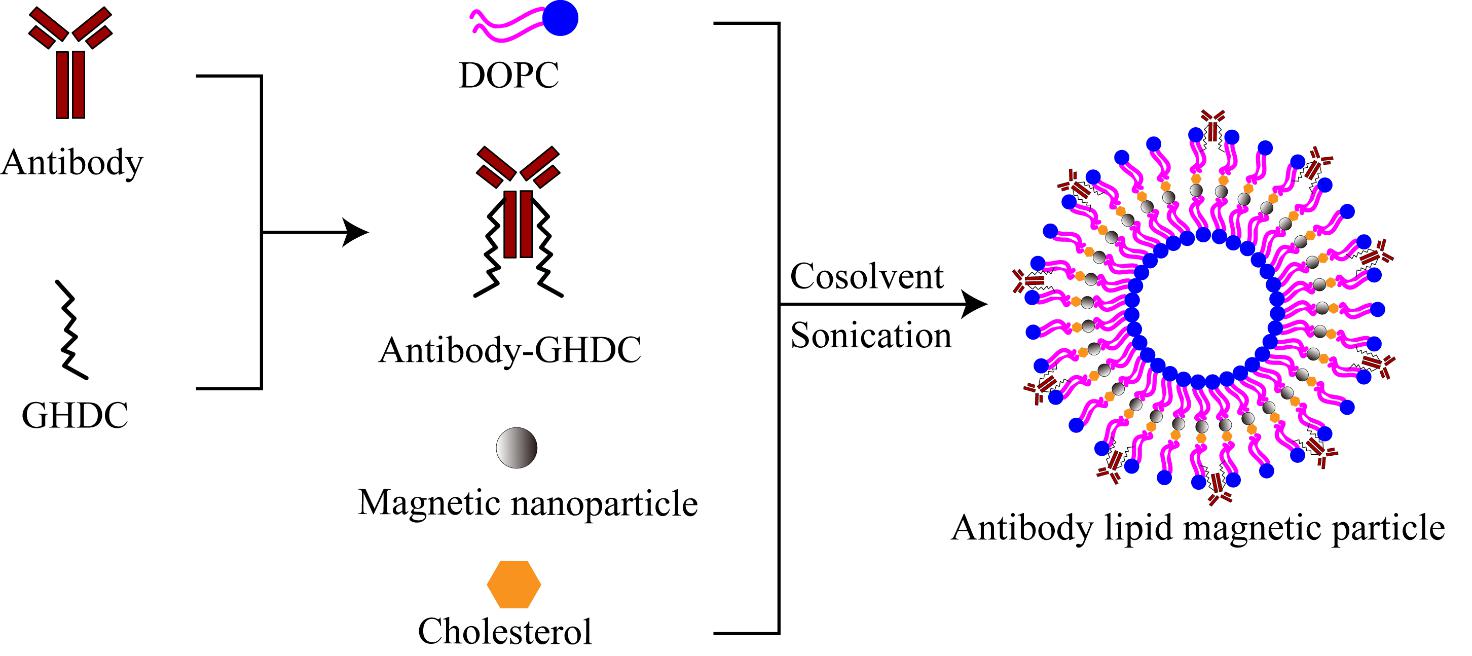

Supplement: Supplementary file 4 — Additional file 4: Figure S4. The scheme of magnetic beads preparation process. [file 12951_2021_818_MOESM4_ESM.jpg]
